# TRPC5 channel participates in myocardial injury in chronic intermittent hypoxia

**DOI:** 10.1016/j.clinsp.2024.100368

**Published:** 2024-05-03

**Authors:** Mengmeng Wang, Wen Wen, Yulan Chen, Sharezati Yishajiang, Yu Li, Zhiqiang Li, Xiangyang Zhang

**Affiliations:** a Department of Hypertension, The First Affiliated Hospital of Xinjiang Medical University, China; b Second Department of General Internal Medicine, The First Affiliated Hospital of Xinjiang Medical University, China; c Laboratory Animal Center, Xinjiang Medical University, China

**Keywords:** Canonical transient receptor potential channel 5, Chronic intermittent hypoxia, Myocardial Injury, Oxidative Stress, Reactive Oxygen Species

## Abstract

**Objective:**

The purpose of this study is to develop an animal model of Chronic Intermittent Hypoxia (CIH) and investigate the role of the TRPC5 channel in cardiac damage in OSAHS rats.

**Methods:**

Twelve male Sprague Dawley rats were randomly divided into the CIH group and the Normoxic Control (NC) group. Changes in structure, function, and pathology of heart tissue were observed through echocardiography, transmission electron microscopy, HE-staining, and TUNEL staining.

**Results:**

The Interventricular Septum thickness at diastole (IVSd) and End-Diastolic Volume (EDV) of rats in the CIH group significantly increased, whereas the LV ejection fraction and LV fraction shortening significantly decreased. TEM showed that the myofilaments in the CIH group were loosely arranged, the sarcomere length varied, the cell matrix dissolved, the mitochondrial cristae were partly flocculent, the mitochondrial outer membrane dissolved and disappeared, and some mitochondria were swollen and vacuolated. The histopathological examination showed that the cardiomyocytes in the CIH group were swollen with granular degeneration, some of the myocardial fibers were broken and disorganized, and most of the nuclei were vacuolar and hypochromic.

**Conclusion:**

CIH promoted oxidative stress, the influx of Ca^2+^, and the activation of the CaN/NFATc signaling pathway, which led to pathological changes in the morphology and ultrastructure of cardiomyocytes, the increase of myocardial apoptosis, and the decrease of myocardial contractility. These changes may be associated with the upregulation of TRPC5.

## Highlights

• The study developed an animal model of chronic intermittent hypoxia (CIH).
• CIH activated oxidative stress (OS) inhearts, increased ROS, and up-regulated TRPC5.
• TRPC5 is related to OS, the imbalance of Ca^2+^ homeostasis, and myocardial injury.

## Introduction

Obstructive Sleep Apnea-Hypopnea Syndrome (OSAHS), which is characterized by recurrent upper airway collapse while sleeping, can cause fragmented sleep, fluctuating intrathoracic pressure, and intermittent hypoxia,^1^ leading to multi-organ and multi-system damage,^2^ or even nocturnal sudden death.^3^ It is estimated that 936-million adult patients aged 30‒69 years suffer from OSAHS worldwide. The number of patients with moderate-to-severe OSAHS is 425 million, of which 18.8 % (176 million) are from China.^4^ OSAHS has a complicated etiology and mechanism, and it is simple to lead to comorbidities. Cardiovascular illness is one of the numerous comorbidities brought on by OSAHS that contributes significantly to adverse events.^4^ Patients with OSAHS may experience angina due to nocturnally time intermittent hypoxia.^5^^,^^6^ Additionally, the precise mechanism causing myocardial injury is unknown, which makes treating OSAHS more difficult. Patients with OSAHS who did not receive timely therapy saw significant reductions in their quality of life and employment. To enhance the quality of life for patients with OSAHS, it is imperative that the mechanism of myocardial injury in OSAHS be thoroughly investigated and that targeted therapeutic medications be developed by this theoretical foundation.

The primary cause of OSAHS and a major contributing factor to cardiovascular disease is Intermittent Hypoxia (IH).^7^ The typical features of OSAHS are better defined by chronic intermittent hypoxia-reoxygenation models, which simulate sleep-induced hypoxic reoxygenation and hypercapnia in patients. Exposure to CIH also generated cardiovascular symptoms that are comparable to those observed in OSAHS patients. Rats are the primary animal model utilized in the study of cardiovascular diseases associated with CIH; as rats are nocturnal and their sleep schedules coincide with human working time, these models are well-suited for studying sleep disorders.

IH results in oxidative stress by reducing oxygen molecules partially and raising intracellular levels of Reactive Oxygen Species (ROS).^8^ Functional crosstalk exists between ROS and Ca^2+^ signaling. Ca^2+^ signaling promotes the production of ROS, the increased ROS causes the opening of Ca^2+^ channels, and finally leads to an imbalance in Ca^2+^ homeostasis.^9^ The canonical transient receptor potential channels are nonselective cation channels mainly involved in the permeation of Ca^2+^.^10^ They are expressed at very low levels in mammalian cardiomyocytes under physiological conditions and may increase during pathological processes.^11^ An earlier study discovered that OSAHS was crucial for myocardial injury. Additionally, the authors discovered that rats with OSAHS had considerably higher levels of TRPC5 protein and mRNA expression in their myocardial tissue,^12^ which was closely associated with myocardial injury.

TRPC5 acts as a nonselective Ca^2+^ channel, and its activation leads to Ca^2+^ influx, which increases cytoplasmic Ca^2+^ concentration.^13^ The Nuclear Factor of Activated T-cells (NFAT) is an important signaling molecule downstream of TRPC5;^14^ NFATC1–4 protein is regulated by Ca^2+^-calcineurin (CaN)-dependent signaling.^15^ Cytoplasmic Ca^2+^ binds to calmodulin to form the Ca^2+^– CAM complex and, in turn, activates CaN, which dephosphorylates NFATc and transfers it to the nucleus.^16^ NFATc can promote the gene transcription of TRPC5 after entering the nucleus,^17^ thus leading to a vicious cycle of this pathological process. Therefore, understanding the role of TRPC5 in CIH-induced myocardial damage is crucial for the development of OSAHS treatment.

## Materials and methods

### OSAHS animal model

All animal experiments were reviewed and approved by the Ethics Committee of Experimental Animals of Xinjiang Medical University (n° IACUC-20,210,326–08) and in accordance with the ARRIVE guidelines. A total of 12 male Specific-Pathogen-Free (SPF) ‒ grade healthy Sprague-Dawley (SD) rats, aged 8–10 weeks, were obtained from the Experimental Animal Center of Xinjiang Medical University.(Keep the environment ventilated, temperature 20°∼24 °C, humidity 40 %∼60 %, light and dark cycle 12 h/12 h), all rats were randomly divided into two groups: the CIH group and the Normoxic Control (NC) group (*n* = 6 in each group). The rats in the CIH group were placed in the intermittent hypoxic chamber (ProOX-100-CIH, TOW-Int Tech, Shanghai, China) from 10 a.m. to 6 p.m. every day for 8 weeks. This chamber was cyclically filled with nitrogen and oxygen, and the gas mixture was extracted (a cycle of 4 min each). In each cycle, the chamber was filled with nitrogen in the first 90 s and then maintained for 30s after the oxygen concentration reached 6.5 %, followed by 60s to discharge the gas in the chamber until the oxygen concentration increased to 21 % and then lasted for 60 s. The rats in the NC group were placed in a chamber and compressed air was continually fed into it to keep the oxygen concentration in the compartment at 21 %. The rats were kept in the same living environment and under the same conditions except for the experimental time every day. All rats were housed in the same environment except during the experiment time.

The rats were anesthetized with ketamine within 24 hours of their last exposure to CIH. After thoracotomy, the rats’ hearts were fully removed, and the apex of the heart was fixed with 2.5 % glutaraldehyde for Transmission Electron Microscopy (TEM) detection. The remaining was divided into four parts: one-fourth was placed in pre-cooled Phosphate-Buffered Saline (PBS) for immediate detection of ROS and Ca^2+^, one-fourth was fixed with 4 % paraformaldehyde for Hematoxylin-Eosin (HE) staining, and terminal deoxynucleotidyl transferase-mediated dUTP Nick-End Labeling (TUNEL), and the remaining one half was frozen rapidly in liquid nitrogen for mRNA and protein detection.

All rats were weighed and anesthetized with ketamine within 24 hours after modeling. Echocardiography was performed using an HD11XE Ultrasonic diagnostic instrument (Phillips, Netherlands) with an S12–4 transducer (frequency 12 MHz). The Left Ventricular (LV) long-axis Two-Dimensional (2D) ultrasound image detection was performed on a parasternal LV long-axis view, and the following parameters were measured using M-mode: Interventricular Septal thickness at end-diastole (IVSd), LV End-Diastolic Volume (EDV), LV Ejection Fraction (LVEF) and LV Fractional Shortening (LVFS).

### TEM

The apical myocardial tissue was promptly cut into 1 mm^3^ cubes after thoracotomy, fixed with 2.5 % glutaraldehyde solution overnight at 4 °C, washed in PBS, and post-fixed in 1 % osmium tetroxide. The samples were dehydrated in gradient ethanol before being embedded in Epon812 resin and then sliced. The ultrathin sections were then double-stained with uranium deoxy acetate-lead citrate. JEM-1230 TEM (Jeol, Japan) was used for observation and imaging.

### HE-staining

After fixation, the heart tissues were dehydrated with gradient ethanol, made transparently, dipped in wax, embedded, and cut into sections of 4 μm thickness. Then, the sections were placed in a 65 °C thermostat for 1.5h–2 h. After dewaxing, hydration, hematoxylin staining, hydrochloric acid ethanol differentiation, and eosin staining, the excess dye was washed off. The sections were dehydrated, made transparent, and sealed with a Permount mounting medium. A Nikon biomicroscope E200 (Nikon, Japan) was used to monitor and image the pathological alterations in cardiac tissue.

### TUNEL staining

The tissue slices of 3 μm thickness were dewaxed, hydrated, inactivated with endogenous peroxidase by antigen repair, and then digested with proteinase K at 37 °C for 15 min. The samples were incubated with the labeling buffer at 37 °C for 2 h and then with blocking buffer at room temperature for 30 min. Then, diluted biotinylated anti-digoxin antibody and the secondary anti-streptavidin-biotin complex were added to the sample slices. After reacting at 37 °C for 30 min, DAB was used for the chromogenic reaction. After re-staining with hematoxylin, the slices were sealed with Permount mounting medium and baked overnight at 37 °C. The cell apoptosis (cells with brownish-yellow granules in the nucleus) was observed and imaged using a Nikon biomicroscope E200. To calculate the apoptosis rates, five fields were chosen at random for each slice.

### Detection of ros

Rat cardiac tissues were washed twice with fresh PBS before being put on a 200-mesh sieve. After gently grinding, the single-cell suspensions were transferred to 1.5 mL centrifuge tubes and centrifuged at 1500 rpm for 5 minutes. After discarding the supernatant, 10 M DCFH-DA was applied to each tube. After mixing and incubating at 37 °C for 30 min, the cells were centrifuged at 1500 rpm for 5 min, collected, and washed with PBS twice. The cells were resuspended in PBS, and an Attune NxT flow cytometer (Thermo Fisher, USA) was used to detect the fluorescence intensity of cells at the excitation wavelength of 488 nm and emission wavelength of 529 nm.

### Detection of Ca^2+^

Fresh rat myocardial tissues were taken and washed twice with PBS. The tissues were placed on a 200-mesh sieve and gently ground. The single-cell suspensions were pulverized and transferred to 1.5-Ml centrifuge tubes, where they were centrifuged at 1500 rpm for 5 minutes. After discarding the supernatant, 1 mL of Fluo-4/AM staining solution with a final concentration of 5 μM was then added to each tube. The tubes were thoroughly mixed and incubated for 15 minutes at 37 °C in an incubator. The cells were collected after centrifugation at 1500 rpm for 5 min and washed with PBS twice. Subsequently, they were resuspended in 500 μL of PBS and detected. The fluorescence intensity was measured using a Thermo Fisher Attune NxT flow cytometer at excitation wavelengths of 488 nm and emission wavelengths of 529 nm.

### Quantitative reverse transcription-polymerase chain reaction

Total RNA was extracted from the rat myocardial tissue using a TRIzol reagent. After reverse transcription into cDNA following the instructions of the Reverse Transcription-Polymerase Chain Reaction (RT-PCR) kit, a fluorescence quantitative real-time PCR was performed to detect the expression of target mRNAs.

The primer sequences were as follows:

Caspase-3 forward: 5′-AATTCAAGGGACGGGTCATG-3′; reverse: 5′-TGACACAATACACGGGATCTG-3′.

TRPC5 forward: 5′-TGCAACTGTGTGGAGTGTGT-3′; reverse: 5′-CACCTTGCTCAGCTCCTTGA-3′.

CaNAα forward: 5′-ATAACGATGGGAAGCCTCGT-3′; reverse: 5′-CAAACTGTGACTGGGGCATC-3′.

NFATc1 forward: 5′-GAAGACTGTCTCCACCACCA-3′; reverse: 5′-CCGATGTCTGTCTCCCCTTT-3′.

NFATc4 forward: 5′-CTGTCAAAGCTGCTCCTGGA-3′; reverse: 5′-GTAGCCACCATCTTGCCAGT-3′.

GAPDH forward: 5′-CAGGGCTGCCTTCTCTTGTG-3′; reverse: 5′-GATGGTGATGGGTTTCCCGT-3′.

The total volume of the reaction system was 20 μL, including the following: Eva Green 2 × qPCR MasterMix, 10 μL; forward primer, 0.6 μL; reverse primer, 0.6 μL; cDNA, 1.0 μL; and RNase-free water, 7.8 μL. The reaction conditions were as follows: pre-denaturation at 95 °C for 10 min, followed by 40 cycles of denaturation at 95 °C for 15 s, and annealing/extension at 60 °C for 60 s. GAPDH was used as the internal reference gene to analyze the melting curve. The 2^−△△^CT method was used to calculate the relative expression of target genes.

### Western blot analysis

The radioimmunoprecipitation assay lysis buffer was used to extract total protein, and the protein concentration was measured using a BCA protein quantification kit. Sodium dodecyl sulfate-polyacrylamide gel electrophoresis was used to separate the proteins, which were then transferred to polyvinylidene difluoride membranes. The membranes were blocked for 1 h using a blocking buffer containing 5 % skimmed milk powder and incubated with the corresponding primary antibodies overnight at 4 °C. The antibody dilution ratios were as follows: mouse anti-caspase-3 antibody (1:400, bsm-33,284 M, Bioss), anti-TRPC5 antibody [S67–15] (1:800. ab240872, Abcam), recombinant anti-calcineurin A antibody [EPR24997–22] (1:400, ab282104, Abcam), anti-NFATc1 antibody (7A6) (1:300, sc-7294, Santa Cruz), anti-NFATc4 antibody (B-2) (1:300, sc-271,597, Santa Cruz), rabbit anti-phospho-NFATc1 (Ser233) antibody (1:300, bs-19785R, Bioss), anti-phospho-NFATc4 (Ser289) polyclonal antibody (1:400, PA5–105,650, Thermo Fisher), and anti-β-actin antibody (1:1000, 100,166-MM10, Sino Biological). The membrane was washed three times with Tris-buffered saline Tween-20 (TBST) the next day and incubated with HRP-labeled secondary antibody (1:5000) for 1 h at room temperature. The membrane was washed three times with TBST. Then, 2 mL of Enhanced Chemiluminescence (ECL) chromogenic solution was mixed and added to the membrane. The protein expression levels were detected using a ChemiScope 3000 mini chemiluminescence instrument (Qin Xiang, China). ImageJ 1.51 software (NIH, USA) was used to scan the bands to determine the grayscale values, and statistical analysis was conducted.

### Statistical analysis

SPSS 25.0 software (SPSS, USA) was used for the statistical analysis. The measurement data were presented in the form of mean standard deviation ($\bar{x}\pm s$). To compare the two groups, an independent-sample *t*-test was used. A statistically significant difference is shown by *p* < 0.05.

## Results

### Changes in cardiac structure and function in rats with CIH

**Fig. 1:**
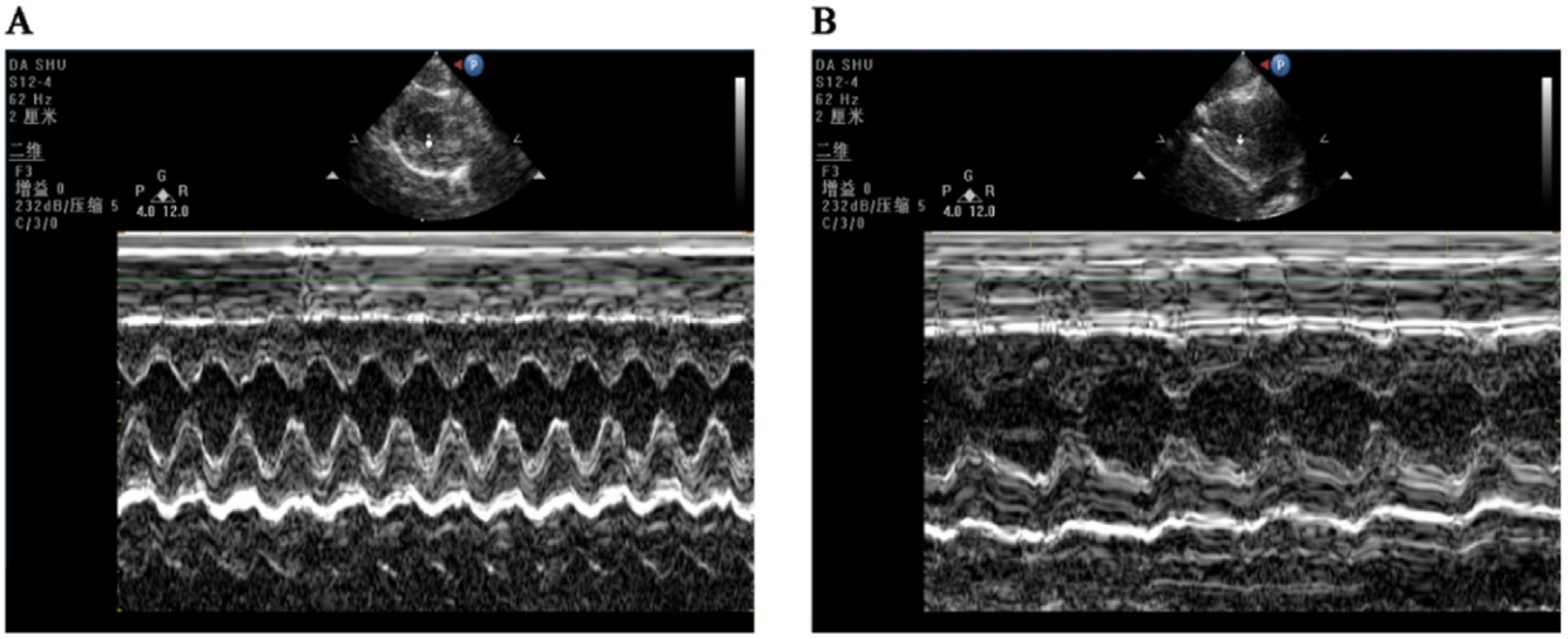
**CIH-induced systolic dysfunction of the left ventricle.** Echocardiographic images of rats in the NC group (A) and CIH group (B).

The IVSd and EDV of rats in the CIH group were significantly higher than those in the NC group ([1.567 ± 0.095] mm vs. [1.250 ± 0.055] mm), *p* = 0.000; [0.397 ± 0.012] mL vs. [0.347 ± 0.099] mL, *p* = 0.000), while LVEF and LVFS significantly decreased in the CIH group compared with the NC group ([68.276 ± 1.260]% vs. [77.827±3.280]%, *p* = 0.001; [38.229 ± 0.908]% vs. [42.689 ± 2.050]%, *p* = 0.001] (Table 1 and Fig. 1).

**Table 1:** Comparison of echocardiographic detection indexes of rats in the two groups.

	NC	CIH	T	p
IVSd (mm)	1.250 ± 0.055	1.567 ± 0.095	−6.635	0.000*
EDV (mL)	0.347 ± 0.099	0.397 ± 0.012	−7.347	0.000*
LVEF (%)	77.827 ± 3.280	68.276 ± 1.260	6.031	0.001*
LVFS (%)	42.689 ± 2.050	38.229 ± 0.908	4.448	0.001*

### Changes in cardiomyocyte morphology, ultrastructure, and apoptosis in rats with CIH

**Fig. 2:**
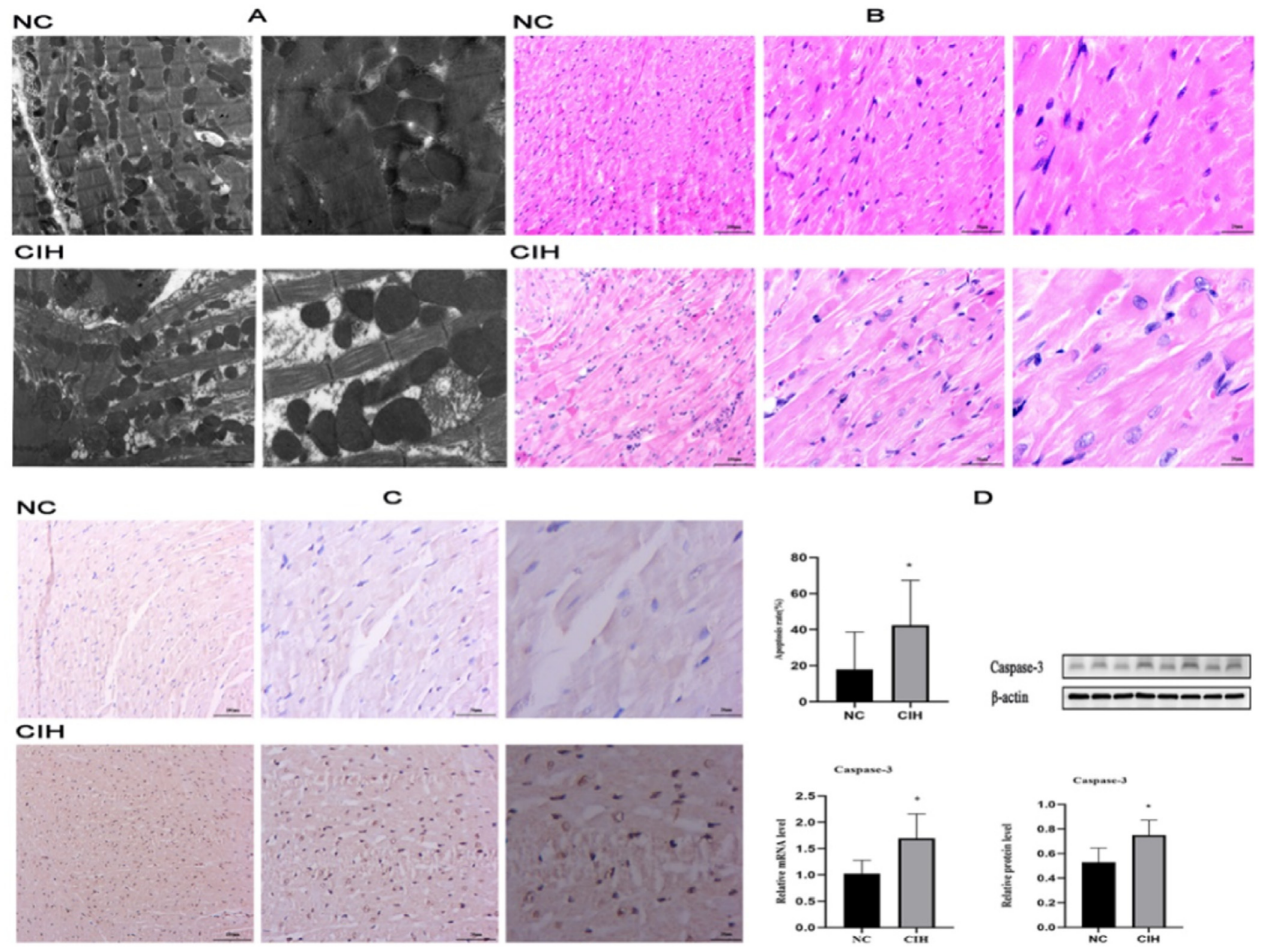
**Morphological and ultrastructural changes and increased apoptosis in cardiomyocytes caused by CIH.** (A) Comparison of myocardial TEM images of rats in the CIH and NC groups. (B) Comparison of myocardial HE-staining images of rats in the CIH and NC groups. (C) TUNEL staining showed that the apoptotic rate of cardiomyocytes in rats with CIH significantly increased. (D) mRNA and protein expression levels of apoptosis-related protein caspase-3 significantly increased in the CIH group (*p < 0.05).

The regular arrangement of myofilaments, consistent sarcomere length, mild dissolution of a small amount of mitochondrial outer membrane, and partially dissolved and flocculent mitochondrial cristae were found in the NC group. The rats in the CIH group had a loose arrangement of myofilaments, varying sarcomere length, dissolved cell matrix, some dissolved and flocculent mitochondrial cristae, dissolved and disappeared mitochondrial outer membrane, the partially swollen and vacuolated appearance of mitochondria (Fig. 2A).

The arrangement, nuclear morphology, and staining of cardiomyocytes in the NC group were normal, while the cardiomyocytes in the CIH group were swollen with granular degeneration. A part of the myocardial fibers was broken and disorganized, and most of the nuclei were vacuolar and hypochromatic (Fig. 2B).

An increase in the number of brown nuclei and a significantly higher rate of apoptosis were observed in the cardiomyocytes in the CIH group compared with the NC group ([41.875 ± 21.034 %] vs. [14.625 ± 16.062]%, *p* = 0.011) (Fig. 2C).

The mRNA and protein expression levels of apoptosis-related protein caspase-3 were significantly higher in the CIH group than in the NC group (*p* = 0.012; *p* = 0.040) (Fig. 2D).

### ROS and Ca^2+^ production increased in the myocardium of rats with CIH

**Fig. 3:**
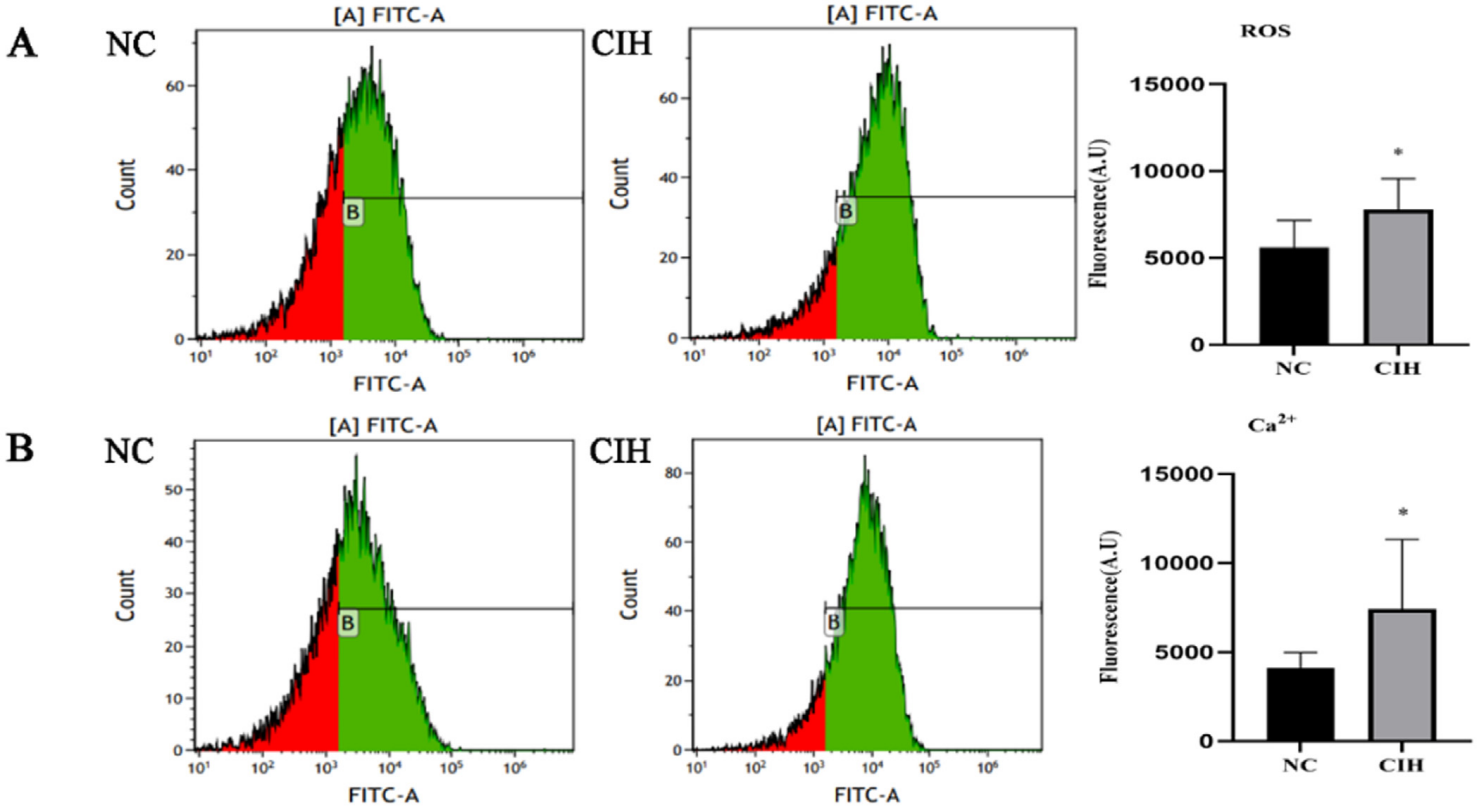
**Increased ROS and Ca^2+^ levels in the myocardium of rats induced by CIH.** (A) ROS fluorescence intensity was significantly higher in the CIH group than in the NC group (*p < 0.05). (B) The fluorescence intensity of Ca^2+^ significantly increased in the CIH group than in the NC group (*p < 0.05).

ROS and Ca^2+^ levels in the myocardium of rats were detected by flow cytometry. The results showed that the fluorescence intensities of ROS (Fig. 3A) and Ca^2+^ (Fig. 3B) were significantly higher in the CIH group than in the NC group ([7783.901 ± 1788.393] vs. [5569.983 ± 1569.545], *p* = 0.021; [7420.254 ± 3913.188] vs. [4115.274 ± 866.928], *p* = 0.035).

### Increased expression of TRPC5 and CaNAα, as well as decreased expression of pNFATc1 and pNFATc4, in myocardial tissue of rats with CIH

**Fig. 4:**
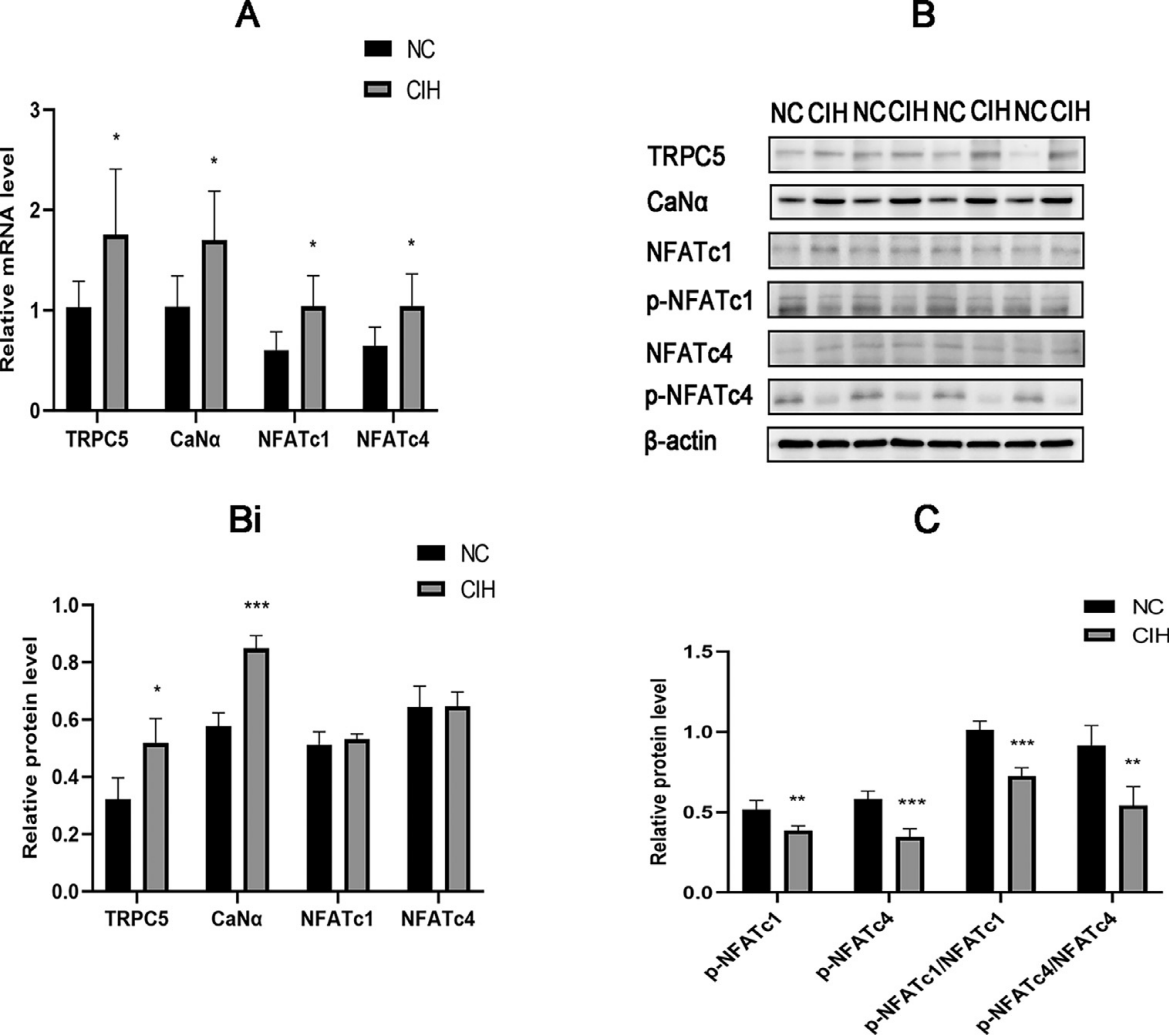
CIH induced high expression of TRPC5 and increased CaN/NFATc. (A) mRNA expression levels of TRPC5, CaNAα, NFATc1, and NFATc4 were significantly elevated in the CIH group (*p < 0.05). (B) Protein expression levels of TRPC5 and CaNAα significantly increased in the CIH group, while no significant difference was found in NFATc1 and NFATc4 protein expression (*p < 0.05, ^⁎⁎⁎^*p* < 0.001). (C) p-NFATc1 and p-NFATc4 expression, p-NFATc1/NFATc1, and p-NFATc4/NFATc4 significantly decreased in the CIH group (^⁎⁎^*p* < 0.01 and ^⁎⁎⁎^*p* < 0.001).

The mRNA of TRPC5, CaNAα, NFATc1, and NFATc4 significantly increased in the myocardial tissue of rats in the CIH group compared with the NC group (*p* < 0.05), At the same time, the expressions of TRPC5 and CaNα proteins in the myocardial tissue of the rats in the CIH group were significantly higher than those in the NC group, while no significant difference was observed in NFATc1 and NFATc4 protein expression (Fig. 4A and 4B).

The protein expression of p-NFATc1 and p-NFATc4 in the myocardial tissue, p-NFATc1/ NFATc1, and p-NFATc4/NFATc4 significantly decreased in the CIH group compared with the NC group (*p* < 0.05) (Fig. 4C).

## Discussion

OSAHS is a risk factor for cardiovascular disease that has been linked to hypertension, arrhythmia, coronary artery disease, and heart failure.^18^ In this study, the CIH model was constructed to simulate OSAHS, which has high stability and good repeatability and simulates the intermittent hypoxia caused by OSAHS from the pathophysiological mechanism.^19^ It simulates intermittent hypoxia caused by OSAHS from a pathological and physiological perspective, making it an ideal model for studying the impact of the hypoxia mechanism in OSAHS on the cardiovascular system and its possible mechanisms. The objective is to offer dependable and efficient experimental data for the clinical management of cardiovascular illnesses associated with OSAHS, as well as for the avoidance and management of consequences.^20^ The authors found that CIH adversely affected LV function. The echocardiographic measurements showed an increase in LVIDs and EDV and a decrease in LVFS and LVEF compared with controls (Table 1 and Fig. 1), indicating a decrease in LV systolic function, which were consistent with the results reported by Wei et al.^21^ Cardiomyocyte injury promoted the development of cardiac dysfunction in rats with OSAHS.^22^ The results of TEM showed that the myofilaments in the CIH group were loosely arranged, the sarcomere length varied, the cell matrix dissolved, some mitochondrial cristae also dissolved and flocculated, the outer mitochondrial membrane lysed and disappeared, and some mitochondria were swollen and showed vacuolar-like changes (Fig. 2A). These findings were consistent with that of Wei et al.^23^ Cardiomyocytes have a vast number of mitochondria, and the majority of the ATP required by the heart (approximately 95 %) is obtained through mitochondrial oxidative metabolism.^24^ Mitochondrial damage can affect the function of cardiomyocytes, resulting in cardiac dysfunction. HE-staining showed swelling of cardiomyocytes in the CIH group with granular degeneration, broken and disorganized cardiac fibers, and vacuolated and hypochromatic nuclei (Fig. 2B), which agreed with the findings of Zhao et al.^25^

IH can cause oxidative stress and increase the production of oxygen radicals, which leads to oxidative damage to the cell structure by initiating lipid peroxidation, protein carbonylation, and DNA oxidation, thus causing irreversible myocardial cell damage and cardiac dysfunction.^26^ TUNEL staining revealed an increase in TUNEL-positive cells and a significantly higher rate of apoptosis in the CIH group compared with the NC group (Fig. 2C). CIH can induce cardiomyocyte apoptosis through the Fas death receptor-induced apoptosis pathway, mitochondria-dependent apoptosis pathway,^27^ and endoplasmic reticulum stress apoptosis pathways. Apoptosis can cause cardiomyocyte loss, thus affecting cardiac function.^28^^,^^29^ The authors also examined the mRNA and protein expression of caspase-3, a key biochemical marker of apoptosis, and found that caspase-3 mRNA and protein expression increased after CIH exposure (Fig. 2D), suggesting enhanced cardiomyocyte apoptosis.

IH resembles ischemia-reperfusion injury.^30^ ATP generation is lowered during hypoxia, and mitochondrial oxidative phosphorylation is inhibited. A huge number of oxygen molecules enter the mitochondria during reoxygenation, producing a large amount of ROS.^31^ ROS triggers cytoplasmic Ca^2+^ overload, leading to an excessive increase in mitochondrial Ca^2+^ levels and causing changes in mitochondrial permeability, ATP loss, and mitochondrial swelling, ultimately resulting in cellular damage.^32^ The authors examined the ROS level in myocardial tissue and found that the ROS level was significantly higher in the CIH group than in the NC group (Fig. 3A), which was consistent with the findings of Moulin et al.^33^ ROS, as a product of oxidative stress, can affect Ca^2+^ levels in the myocardial tissue by regulating plasma membrane and intracellular Ca^2+^ channels as well as Ca^2+^ transporters.^34^ The authors also found that the levels of Ca^2+^ in the myocardial tissue were significantly higher in the CIH group compared with the NC group (Fig. 3B). TRPC5, as a permeable cationic channel of Ca^2+^ ions, can be activated by oxidative stress.^35^ The earlier investigation found that IH elevated TRPC5 mRNA and protein expression levels in cardiac tissue,^12^ implying that TRPC5 may be implicated in myocardial damage during IH.

TRPC5 is found in a variety of organs, including the brain,^36^ kidney, and cardiovascular system.^37^ TRPC5′s significance in cardiovascular disease has received a lot of attention in recent years. Liu et al. found that the mRNA and protein levels of TRPC4 and TRPC5 significantly increased in rats with spontaneous hypertension compared with controls. Also, the mid-wall FS was lower in rats with spontaneous hypertension than in controls, suggesting that these channels might be involved in developing LV systolic dysfunction.^38^ Urocortin-2 attenuates adverse cardiac remodeling induced by ischemia-reperfusion injury by inhibiting the expression of TRPC5 and Orai1 and their interactions.^39^ The activation of TRPC6 and TRPC5 inhibits re-endothelialization of arterial injury in mice with hypercholesterolemia.^40^ The qRT-PCR and Western blotting results showed that the mRNA and protein expression levels of TRPC5 significantly increased in rats with CIH compared with controls (Fig. 4A and B), the authors confirmed the increased production of oxidative stress products ROS and Ca^2+^ in cardiomyocytes during IH (Fig. 3A), meanwhile, the expression of TRPC5 increased. Therefore, it was reasonable to speculate that ROS activated TRPC5^35^ and further caused Ca^2+^ influx, resulting in an increased cytoplasmic Ca^2+^ concentration, which was involved in myocardial injury.^41^

NFATc, as a downstream of the TRPC5 channel, is regulated by the calcium- and calcineurin-dependent signaling pathway.^42^ In dormant cells, NFATc family members are usually located in the cytoplasm and exist in hyperphosphorylated forms. After activation of CaN, NFATc proteins are directly dephosphorylated and translocated into the nucleus to promote the expression of related genes.^43^ The authors identified the mRNA and protein expression of the CaN subunit CaN, and we also discovered that CaN mRNA and protein expression levels rose in the CIH group's cardiac tissues (Fig. 4A and B). The mRNA expression of NFATc1 and NFATc4 in the myocardial tissue increased (Fig. 4A), While expression levels of NFATc1 and NFATc4 proteins were not significantly different between the two groups (Fig. 4B), which might be related to post-translational protein modification. Despite no difference in the protein expression of NFATc1 and NFATc4, the levels of p-NFATc1 and p-NFATc4 were significantly reduced in the CIH group compared with the NC group (Fig. 4C). It was speculated that IH promoted the activation of NFATc1 and NFATc4 transcription factors, which was indicated by reduced p-NFATc1/NFATc1 and p-NFATc4/NFATc4. These conclusions were consistent with the activation of the NFATc transcription pathway. As TRPC5 is the target gene of NFATc, the nuclear entry of NFATc may lead to increased TRPC5 gene transcription,^17^ causing an increase in the TRPC5 protein level, which leads to more Ca^2+^ entry and accelerates the vicious cycle of this pathological process.

In summary, the authors successfully constructed an OSAHS rat model and also confirmed by multiple detection methods that CIH changed the morphology and ultrastructure of rat cardiomyocytes ‒ the dissolution of the cell matrix, and the apoptosis of cardiomyocytes, in which TRPC5 played a key role. CIH increased the levels of ROS in the myocardial tissue, thus opening TRPC5 and increasing Ca^2+^ influx, which leads to an imbalance of Ca^2+^ homeostasis, the activation of the CaN/NFATc signaling pathway, and the promotion of myocardial injury.

## Limitations

This study was not performed to reverse verify the effect of TRPC5 on myocardial injury and the effect of ROS on TRPC5. The mechanism of action of TRPC5 in myocardial injury will be investigated in the future by the knockout of the TRPC5 gene, the use of ROS inhibitors in vitro, and the up-regulation and downregulation of TRPC5.

## Ethics approval

All animal experiments were reviewed and approved by the Ethics Committee of Experimental Animals of Xinjiang Medical University.

## Declaration

The corresponding author submitted the manuscript on behalf of all the authors. The authors declare no competing interests.

## Authors’ contributions

The experimental operation of this study was mainly completed by MMW, WW, SRZTY and ZQL. The paper was written by MMW and WW. YLC and YL guided the overall idea of the study. YLC put forward valuable opinions on the writing of the article. XYZ provided a critical reading of the manuscript.

## Declaration of competing interest

The authors declare no conflicts of interest.
